# Celebrating 50 years of penile implants

**DOI:** 10.1038/s41443-023-00663-y

**Published:** 2023-01-17

**Authors:** Steven K. Wilson, Martin S. Gross

**Affiliations:** 1 Department of Urology, Institute for Urologic Excellence, La Quinta, CA USA; 2 Section of Urology, Dartmouth-Hitchcock Medical Center, Lebanon, NH USA

**Keywords:** Erectile dysfunction, Sexual dysfunction

## The beginning

**Fig. 1:**
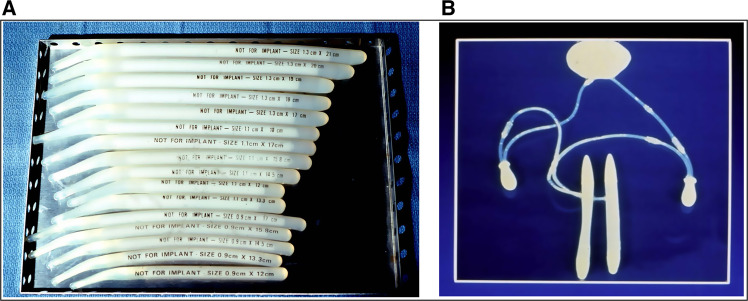
The first prosthetic devices. **A** Small-Carrion prosthesis. **B** Early Scott (later AMS) prosthesis.

Many prosthetic urologists are not aware that the first semi-rigid penile prosthesis and the first three-piece inflatable penile prosthesis (IPP) were introduced in the United States (US) almost simultaneously 50 years ago. The Small-Carrion semi-rigid prosthesis, created by urologists Michael Small and Hernan Carrion, consisted of paired silicone rods of varying lengths (Fig. 1A) which were implanted via a perineal incision [1]. The first three-piece inflatable prosthesis (Fig. 1B) invented by urologist F. Brantley Scott was surgically placed through a large abdominal incision that stretched from pubic bone to umbilicus [2].

**Fig. 2:**
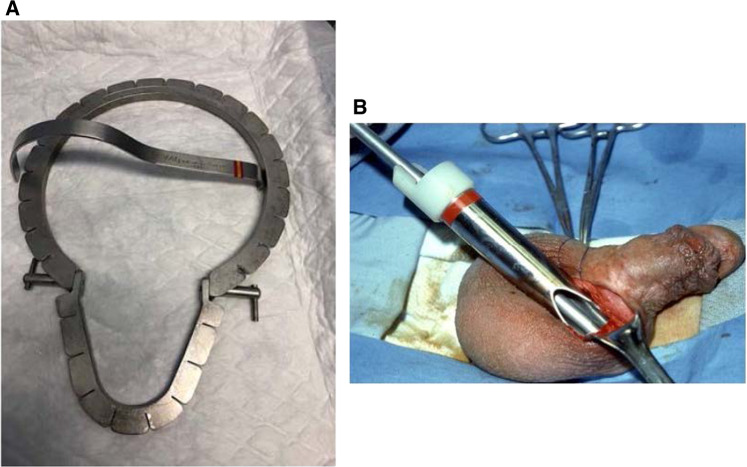
Instruments to facilitate penoscrotal placement. **A** Original Scott metal retractor. **B** Scott reservoir insertion tool.

It took Dr Scott several years to formulate a new incision in the upper scrotum. At the time of the penoscrotal incision’s introduction in the early 1980s, Dr Scott also introduced a metal retractor (Fig. 2A) and reservoir inserter (Fig. 2B). Mentor, a silicone breast implant manufacturer, entered the market in 1983 with a new three-piece IPP. This device closely resembled the Scott prosthesis (then called AMS for American Medical Systems) [3]. The device was so similar that subsequent litigation forced Mentor to pay a commission to AMS on each Mentor IPP sold for many years. But this similarity also precluded the FDA from requesting clinical studies to bring the new IPP to market. Eventually, the FDA changed its mind and in the mid-1990s required Mentor to perform a pre-market approval clinical investigation in a prospective manner. This was despite the fact the Mentor three-piece inflatable had been implanted in US patients by urologists for over a decade [4].

Meanwhile, the surgical incision to implant rods rapidly changed from perineal to penoscrotal for the Small-Carrion and a plethora of competitive devices quickly appeared on the marketplace. These new devices featured two enhancements. The rods were made trimmable, reducing the need for the myriad of lengths of the Small-Carrion implant. Metal wires were also embedded in the intracorporal rods which markedly improved concealment. The presence of the metal wire insert allowed the device’s nomenclature to change from “semi rigid” to “malleable.” Current semi-rigid devices have changed very little since these two enhancements in the early 1980s. 1993 was the first year IPPs passed the malleables in number implanted per year; in 2015 it was noted that the percentage of inflatables compared to malleables had increased twelvefold in the last 10 years [5]. It is believed that in 2020, the number of malleables sold in the US was 1500 - as compared to 30,000 IPPs [6].

## Reliability issues and device adaptations

**Fig. 3:**
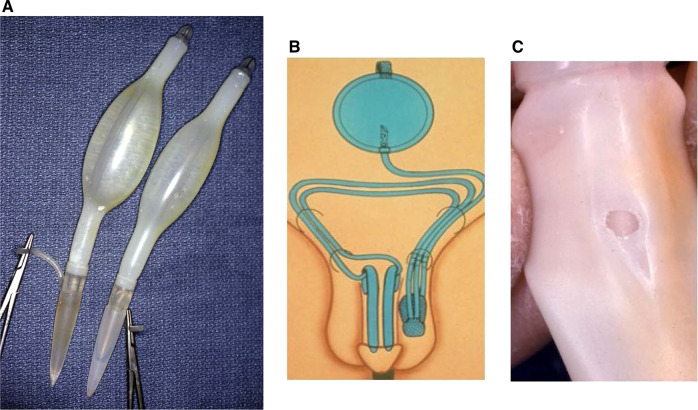
The first Scott three-piece inflatable prosthesis. **A** First silicone cylinders. **B** Diagram of infrapubic implant. **C** Cylinder wear and leak.

The Scott three-piece (now called AMS700) was notoriously unreliable during the 1970s and 1980s. The silicone balloon cylinders (Fig. 3A) were prone to aneurysm formation and leakage. The three-piece IPP was widely disparaged by urologists and former patients who were re-rendered impotent again by device failure (Fig. 3B). In addition to poor device survival, the large infrapubic incision was troublesome and the surgery very time consuming as the non-kink-proof tubing required passing the tubing through both inguinal canals (Fig. 3C). The IPP’s reliability and the frequency of reoperation created a demand for other inflatable devices that were simpler to implant, more reliable, and did not require the dreaded reservoir insertion.

The Hydroflex IPP was introduced in 1985. It consisted of a pair of liquid rods that had a non-distensible chamber filled with 3 cc of saline upon pumping the tip of the rod. The Hydroflex was initially a popular choice for both physicians and impotent patients [7] because the surgery was much simpler, and the device was reasonably reliable. Unfortunately, erection rigidity and penile girth with the Hydroflex, and its similar successor the Dynaflex, were significantly compromised because of the small amount of fluid in the reservoir. Today’s descendent of these unitary inflatables is the Ambicor. The Ambicor is a two-piece inflatable consisting of a pump and two liquid rods. Over the years many other unitary and two-piece inflatables appeared and withdrew from the American marketplace (Uniflate, Flexiflate, Mark II, GFS, etc.). None of these devices matched the three-piece in rigidity or flaccidity and now only the Ambicor remains, but it has only a tiny share of the market [8].

**Fig. 4:**
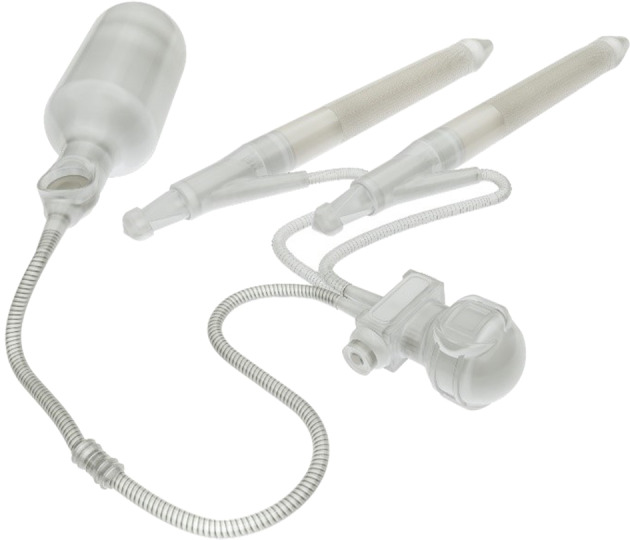
Rigicon Infla10® three-piece prosthesis.

Mentor was eventually purchased by Coloplast (Minneapolis, MN) and AMS by Boston Scientific (Marlborough, MA). Both parent companies continue the historical legacy of five decades of technological advancement of their IPPs. On the 50th birthday of penile implants, a new three-piece IPP developed by a start-up manufacturer is presently going through its FDA-required pre-market approval investigation (Fig. 4). This new American-manufactured IPP is called the Rigicon Infla10® (Rigicon, Ronkonkoma, NY) [9]. Its inventor is Dr Hüseyin Luleci, a Turkish prosthetic urologist who Dr Wilson trained surgically 35 years ago. Dr Luleci has been implanting large volumes of three-piece prostheses and sphincters for decades in Turkey. Upon initial evaluation, the Rigicon Infla10® has similar freedom from mechanical problems as the existing devices [9] and makes use of advantages and enhancements that would appeal to cognizant implanters. These include increasing girth expansion in longer cylinder models and elimination of the problematic “boot” on the cylinder input tubing even though the silicone cylinders are fabricated in layers similar to Boston. Rigicon also manufactures an artificial urinary sphincter awaiting FDA clinical trials and a malleable which is currently available in the US.

## Important enhancements to the IPP

In 2007, a large single-surgeon series of 2304 first-time patients receiving both AMS and Mentor three-piece IPPs concluded the 5-, 10- and 15-year survival from revision surgery to be superior to any other implant utilized in the US. That meant that compared to breast, hip, knee, lens, heart valve and other implants, the IPP was least likely to require revision for medical problem, infection or mechanical breakage [10]. What a comeback today’s IPP has made from the original Scott prosthesis! Causes for the reduction in corrective revision surgery included the many mechanical enhancements to AMS and Mentor three-piece IPPs and the simplification of surgical techniques over the decades. While the mechanical enhancements over the years are too many to list in this editorial, three stand out as paradigm changers.

1. Cylinder rigidity improvement.
2. Device infection reduction.
3. Device lock-out valves.

### Cylinder rigidity

The original Scott cylinders were simply silicone balloons (Fig. 3A). Device inflation created a turgid penis without axial rigidity. Thus, patients had increased girth but floppy penises. This changed after the introduction of the Mentor three-piece IPP in 1983. The Mentor cylinders were constructed of Polyurethane which when filled with saline had excellent rigidity. AMS responded in 1986 by changing cylinder fabrication to two layers of silicone with fabric sandwiched between them. The fabric was similar to wet suit material and resulted in a controlled expansion with excellent rigidity. The rigidity of the erection of both the sandwich construction AMS and Mentor’s cylinders allowed simplification of the treatment for Peyronie’s patients who suffered from impotence. Utilization of the erect and now more rigid cylinders as a fulcrum allowed the physician to model the curvature and regain a straight penis [11]. Modeling represented a new avenue for correcting concomitant erectile dysfunction (ED) and Peyronie’s disease, particularly for urologists less comfortable with elevating the neurovascular bundle and resecting the plaque.

### Device infection reduction

At the dawn of the twenty-first century, AMS introduced the first antibiotic coating of IPP components. AMS coated the entire device except Rear Tip Extenders with Rifampin and Minocycline. Mentor responded in 2002 by applying a hydrophilic coating to the entire Alpha 1. This allowed the physician to dip the device in an antimicrobial solution which then eluted off the device upon implantation. Infection-retardant coating was truly a transformational enhancement. Infection rates plummeted from over 4% in first-time implants to less than 1% [12]. Coincident with the introduction of infection-retardant-coated IPPs, Henry et al. pioneered a technique to significantly reduce revision surgical infections. Revision washout involved complete component exchange (instead of single damaged component replacement) coupled with extensive antiseptic lavage of the implant component spaces [13]. Dariouche also improved surgical site antisepsis by proving the superiority of Chlorhexidine in alcohol to the time-honored skin preparation with Betadine® [14].

The infection reduction in this era of coated implants, when Dr Wilson was performing 300 IPPs per year, was almost palpable. Before the availability of coated devices, Dr Wilson averaged one infected IPP per month (4%). He dreaded going to the office at the end of the month if the infection had not yet presented. For the last 20 years, Dr Wilson has not had a single first-time IPP become infected. In recent years Dr Wilson’s practice has become focused on fixing train wrecks, both his and for other surgeons. Despite this, in Dr Wilson’s tertiary practice of predominantly prosthetic reoperations, he has sustained only two infections in recent memory. Both of these patients had multiple reoperations of their prosthetic prior to their infection. We have learned the hard way that by the fifth redo the odds of infection are basically 100% [15].

### Lock-out valves

The rare complications of reservoir placement in the retroperitoneum can be life-threatening. In our opinion, reluctance to perform reservoir placement is the reason why only 24% of urologists perform one or more IPP per year and why high-volume surgeons perform a disproportionate number of implant surgeries [5]. Mentor invented the lock-out valve in 2000 [16] and AMS followed in 2002. These innovations reduced autoinflation and they also created an opportunity for non-traditional locations for the reservoir. Both manufacturers followed with flatter reservoir designs so they would be less visible and palpable in the abdominal wall. High-volume physicians have embraced the improved safety of ectopic locations for IPP reservoirs [17]. Unfortunately, however, the blind insertion of a long clamp into the layers of the abdominal wall remains frightening to the occasional implanter. As a result, the number of prosthesis surgeries remains skewed toward more implants placed by the most experienced surgeons than by novice implanters [5]. We strongly believe that improved teaching of ectopic placement of IPP reservoirs, perhaps utilizing an anatomic model, would increase mainstream urologist participation in prosthetics above the present 24%. It confounds us that implant manufacturers have failed to follow this logic.

## Improvements in device insertion surgical technique

We have described above how human ingenuity has improved the penile implant. Device modifications, experienced surgeons and careful attention to preventing infection have improved the medical outcomes to “top of the class” for medical devices implanted in men [10]. One would think advances in surgical technique would have diminished. Yet even after 50 years, IPP surgery remains a hotbed of innovation. In the last 15 years, for example, the new subcoronal incision for IPP insertion was first invented by Paulo Egydio, a Brazilian urologist, and was first published in 2016 by Valenzuela’s group [18]. It has not supplanted penoscrotal or infrapubic insertion as yet, but a single surgeon in Seoul, South Korea, has utilized this incision to implant 891 IPPs under local anesthesia [19].

We have described how the IPP’s inventor, Dr Scott, despaired of the abdominal incision and promoted the penoscrotal approach in the early 1980s. By the year 1990 and for 20 years thereafter, 80% of the IPPs in US were performed through a scrotal incision. Penoscrotal implantation was so dominant that many small market distributors did not even stock infrapubic models. On proctoring visits around the world, Dr Wilson was frequently implored not to mention the possibility of infrapubic implantation. Around 20 years ago a young urologist opened a shop in Coral Gables, Florida and almost single-handedly swung the pendulum back to infrapubic. Paul Perito called his technique the minimally invasive inflatable penile implant [20, 21]. He has performed thousands of infrapubic implantations and taught hundreds of surgeons to perform his technique. A typical training day with Dr Perito consists of 8–16 IPPs done through a 2 cm infrapubic incision in less than 15 min skin to skin.

The penoscrotal incision has also advanced over time. Dr Scott invented the penoscrotal incision and his retractor to help exposure; Dr Wilson has enhanced and embellished both. The exposure is improved by making a high transverse scrotal incision. Dr Wilson transformed the reusable metal Scott retractor into the disposable Wilson (Coloplast) and SKW scrotal (Boston Scientific) retractors. By deploying the disposable retractor kits properly, the penoscrotal implant can easily be done in 30 minutes.

Dr Wilson performed the first IPP in the state of Arkansas in 1974 after attending Dr Scott’s second implant workshop. He has learned the following caveats from thousands of first-time implants and thousands more revision surgeries in 50 years of practice [22].

1. Always use a closed suction drain. Exit the drain next to the pump. The puncture will serve as a drainage port for late-developing scrotal hematoma [22].
2. The best dressing is the Henry Mummy Wrap, which may also reduce the likelihood of infection [23].
3. The safest maneuver for correction of curvature with IPP is modeling with the protection of the fossa navicularis by the “chicken choke” maneuver of Perito [24].
4. Copiously irrigate … as Mulcahy says “dilution is the solution to the pollution”.
5. The last person who thinks the patient needs corrective surgery is the original surgeon. When trouble brews, pick up the phone and get another opinion.
6. The first implant has the best chance of a happy outcome. If things go south, abort and come back another day.
7. When in doubt, take it out. This is not life-threatening illness … it’s only impotence.
8. Never rush a revision. “Tincture of time may put a poultice on the patient’s frustration” and gives time for the capsule to form which can be used in the repair [22].
9. There are only two true emergencies demanding an immediate return to surgery: glans ischemia and an incision draining blood, urine, feces or pus [22].
10. Never implant a stranger. Get to know your patient. “Not everybody who wants an implant should have one”.

## The penile implant’s last frontier: cosmetic enhancement of the flaccid penis

A constant query from patients preparing for a penile implant is, “Doc, can you make it bigger?” This question is totally different when from our typical impotent 65-year old wishing for surgical correction of his ED than from a fully potent 35-year old. This latter individual is seeking help with the size of his flaccid penis which functions just fine with sexual arousal. Many of these patients have a penis that is statistically normal in size. In my opinion, this young potent patient obsessed with his penile appearance represents the last frontier for penile implants to achieve acceptance by the urologic community.

The situation the patient with penile dysmorphia faces today is similar to that faced by the older ED patient contemplating IPP in the 1980s. At that time IPP placement was a complex operation with frequent medical complications and the virtual certainty of mechanical breakage. It was decried by traditional urologists. Many of whom, at that time, believed ED was “all in your head.” As we have seen, device enhancements, reduced infections and improved surgical techniques have resulted in the three-piece IPP dominating the inflatable market. Modern IPPs have also displaced the malleable devices and become a well-accepted treatment modality among urologists.

**Fig. 5:**
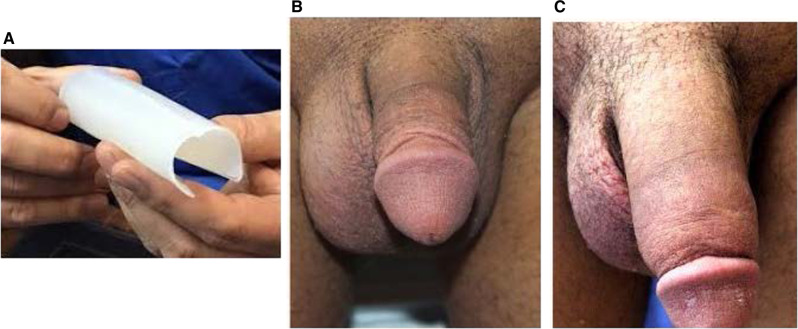
Penuma® penile implant. **A** Penuma® penile implant. **B** Penis before implant. **C** Penis after implant.

The only penile implant presently cleared by the FDA for cosmetic (not functional) improvement of the penis is the Penuma® [25] (Fig. 5A). The device is a soft medical-grade silicone implant surgically inserted subcutaneously to provide cosmetic size enhancement of the flaccid penis. Over 5000 devices have been implanted in the US over the last 17 years. In its infancy, as was the case with the IPP, complications occurred periodically, and revision operations were often necessary. When the complication presented, early surgeons tended to err in preserving the implanted status, sometimes resulting in penile deformity due to scarring when it was eventually explanted.

These complications caused mainstream urology to question the Penuma® penile implant’s reputation. As time has passed, however, improved surgical techniques including a new insertion through a scrotal incision have resulted in quicker surgical insertion with fewer issues [25]. The manufacturer (International Medical Devices, Beverly Hills CA) has also made device enhancements to improve the reliability of a good surgical outcome. Most importantly, contemporary practice has tended towards removing the prosthesis earlier when trouble appears, which diminishes penile scarring and shortening [25, 26].

Recent studies show that today’s Penuma® patients receive 4.8 cm improvement in girth and 2 cm increase in length of the visible flaccid penis (Fig. 5B, C). Self-confidence, self-esteem and satisfaction are improved amongst these challenging, peno-centric patients [25–27]. The latest study shows improved prosthesis survival with less than 10% of patients requiring implant removal for adverse events or patient dissatisfaction [27]. The improved safety and efficacy achieved with contemporary Penuma® implantations hopefully will persuade our colleagues in urology to reassess the use of a subcutaneous implant to improve the cosmetic appearance of the penis…. just as it took decades for us to change our minds about treating impotence with the IPP.
