# Correction to: Celebrating 50 years of penile implants

**DOI:** 10.1038/s41443-023-00691-8

**Published:** 2023-04-03

**Authors:** Steven K. Wilson, Martin S. Gross

**Affiliations:** 1 Department of Urology, Institute for Urologic Excellence, La Quinta, CA USA; 2 Section of Urology, Dartmouth-Hitchcock Medical Center, Lebanon, NH USA

**Keywords:** Erectile dysfunction, Sexual dysfunction

Correction to: *International Journal of Impotence Research* 10.1038/s41443-023-00663-y, published online 17 January 2023

The article “Celebrating 50 years of penile implants”, written by Steven K. Wilson and Martin S. Gross, was originally published electronically on the publisher’s internet portal on 17 January 2023 without open access. With the author(s)’ decision to opt for Open Choice the copyright of the article changed on 11 March 2023 to ©The Authors 2023 and the article is forthwith distributed under a Creative Commons Attribution 4.0 International License, which permits use, sharing, adaptation, distribution and reproduction in any medium or format, as long as you give appropriate credit to the original author(s) and the source, provide a link to the Creative Commons licence, and indicate if changes were made. The images or other third party material in this article are included in the article’s Creative Commons licence, unless indicated otherwise in a credit line to the material. If material is not included in the article’s Creative Commons licence and your intended use is not permitted by statutory regulation or exceeds the permitted use, you will need to obtain permission directly from the copyright holder. To view a copy of this licence, visit http://creativecommons.org/licenses/by/4.0.

